# Characterization of a Prefusion-Specific Antibody That Recognizes a Quaternary, Cleavage-Dependent Epitope on the RSV Fusion Glycoprotein

**DOI:** 10.1371/journal.ppat.1005035

**Published:** 2015-07-10

**Authors:** Morgan S. A. Gilman, Syed M. Moin, Vicente Mas, Man Chen, Nita K. Patel, Kari Kramer, Qing Zhu, Stephanie C. Kabeche, Azad Kumar, Concepción Palomo, Tim Beaumont, Ulrich Baxa, Nancy D. Ulbrandt, José A. Melero, Barney S. Graham, Jason S. McLellan

**Affiliations:** 1 Department of Biochemistry, Geisel School of Medicine at Dartmouth, Hanover, New Hampshire, United States of America; 2 Vaccine Research Center, National Institute of Allergy and Infectious Diseases, National Institutes of Health, Bethesda, Maryland, United States of America; 3 Centro Nacional de Microbiología and CIBER de Enfermedades Respiratorias, Instituto de Salud Carlos III, Majadahonda, Madrid, Spain; 4 MedImmune Inc., Gaithersburg, Maryland, United States of America; 5 AIMM Therapeutics, Academic Medical Center, Amsterdam, Netherlands; 6 Electron Microscopy Laboratory, Cancer Research Technology Program, Leidos Biomedical Research, Inc., Frederick National Laboratory for Cancer Research, Frederick, Maryland, United States of America; Duke University Medical Center, UNITED STATES

## Abstract

Prevention efforts for respiratory syncytial virus (RSV) have been advanced due to the recent isolation and characterization of antibodies that specifically recognize the prefusion conformation of the RSV fusion (F) glycoprotein. These potently neutralizing antibodies are in clinical development for passive prophylaxis and have also aided the design of vaccine antigens that display prefusion-specific epitopes. To date, prefusion-specific antibodies have been shown to target two antigenic sites on RSV F, but both of these sites are also present on monomeric forms of F. Here we present a structural and functional characterization of human antibody AM14, which potently neutralized laboratory strains and clinical isolates of RSV from both A and B subtypes. The crystal structure and location of escape mutations revealed that AM14 recognizes a quaternary epitope that spans two protomers and includes a region that undergoes extensive conformational changes in the pre- to postfusion F transition. Binding assays demonstrated that AM14 is unique in its specific recognition of trimeric furin-cleaved prefusion F, which is the mature form of F on infectious virions. These results demonstrate that the prefusion F trimer contains potent neutralizing epitopes not present on monomers and that AM14 should be particularly useful for characterizing the conformational state of RSV F-based vaccine antigens.

## Author Summary

Respiratory syncytial virus (RSV) causes significant morbidity and mortality in children, yet an efficacious vaccine remains unavailable. Antibodies that preferentially recognize the prefusion conformation of the fusion (F) glycoprotein, particularly those that bind antigenic site Ø at the membrane-distal apex, potently neutralize infection and have aided vaccine design. Here we characterize AM14, a potent human antibody, which we show recognizes a novel epitope midway between the membrane-proximal region and the apex of the prefusion F trimer. The epitope is evenly distributed across two protomers, causing AM14 to be uniquely trimer-specific and, surprisingly, cleavage-dependent. These results indicate that the prefusion trimer is antigenically distinct from the monomer. Our findings also demonstrate that epitopes other than site Ø can be the target of extremely potent neutralizing antibodies and thus provide a new target for structure-based vaccine design. Recognition of this novel epitope could make AM14 an ideal candidate for strategies that combine passive prophylaxis with vaccination, since binding of AM14 would not block elicitation of antibodies against site Ø. Due to its unique specificity, AM14 will also be valuable for probing the conformation of RSV F-based vaccine antigens designed to be in the furin-cleaved trimeric prefusion conformation.

## Introduction

Respiratory syncytial virus (RSV) is a ubiquitous paramyxovirus that infects nearly all children in the U.S. by two years of age [1]. In infants and young children RSV can cause acute lower respiratory tract infections, leading to bronchiolitis and pneumonia. In 2010, RSV was estimated to cause the deaths of more than 200,000 children, accounting for 2.3% of neonatal and 6.7% of infant deaths worldwide [2]. Although infant mortality in the U.S. due to RSV is low, the cost of hospital care for infected infants is estimated to be as high as $750 million per year [3, 4]. Prophylaxis with the humanized monoclonal antibody palivizumab (Synagis) is the only viable intervention for RSV but is limited to use in high-risk infants due to its cost and modest efficacy [5–7]. The development of very potent antibodies or an efficacious vaccine would bring protection to more children and reduce the financial burden of RSV.

Most RSV vaccine candidates contain at least one of the two viral surface glycoproteins: the fusion protein (F) and the attachment protein (G). Of these, only F is absolutely required for infection [8], and it is the target of palivizumab as well as the majority of neutralizing activity in human sera [9–12]. RSV F is a class I fusion protein that is initially synthesized as an inactive precursor. Proteolysis by a furin-like protease at two sites liberates a 27-amino-acid glycopeptide [13–15]. The N- and C-terminal polypeptides, F_2_ and F_1_, respectively, are connected by two disulfide bonds to form a single protomer [16–18]. Although three protomers eventually associate to form the mature trimeric F protein, the order and timing of F protein cleavage and trimerization are unknown.

To facilitate virus entry, the mature F protein is triggered to undergo a transition from a metastable prefusion conformation to a stable postfusion conformation, resulting in fusion of the viral and host-cell membranes. Crystal structures of both prefusion and postfusion F have recently been solved, providing molecular insight into this dramatic structural rearrangement [17–19]. Upon triggering, multiple secondary structure elements in the F_1_ N-terminus assemble into a long alpha helix that extends toward the target cell. This reorganization pulls the hydrophobic fusion peptide from the interior of the trimer and thrusts it into the cellular membrane, resulting in a prehairpin intermediate. The F_1_ C-terminus then migrates toward the N-terminus, irreversibly forming a six-helix bundle and driving fusion of the two membranes [20]. This rearrangement of F can also occur spontaneously, resulting in an abundance of postfusion F on the viral membrane [21].

RSV-neutralizing antibodies that bind both the pre- and postfusion conformations of F were the first to be isolated. These include antibodies 101F and the murine precursor of palivizumab, antibody 1129 [9, 10, 18, 22]. More recently, extremely potent antibodies that specifically target prefusion F have been characterized [19, 23, 24]. Three such antibodies—D25, AM22 and 5C4—all bind to the apex of the prefusion trimer at antigenic site Ø, which is dramatically reorganized during fusion [19]. Antibody MPE8, which cross-neutralizes four pneumoviruses, competes with palivizumab, yet preferentially recognizes the prefusion conformation [25]. In addition to their potential for passive prophylaxis, these antibodies were critical to the design of vaccine antigens stabilized in the prefusion conformation, which were shown to elicit much higher neutralizing activity than postfusion F in mice and rhesus macaques [26].

Although site Ø antibodies were originally thought to recognize quaternary epitopes on the prefusion trimer, they have subsequently been shown to react with monomers [27]. Here we characterize AM14, a potent RSV neutralizing antibody that was previously isolated from human PBMCs [23], and show that it recognizes a novel quaternary epitope on the native trimeric prefusion conformation of RSV F.

## Results

### AM14 potently neutralizes RSV

**Fig 1:**
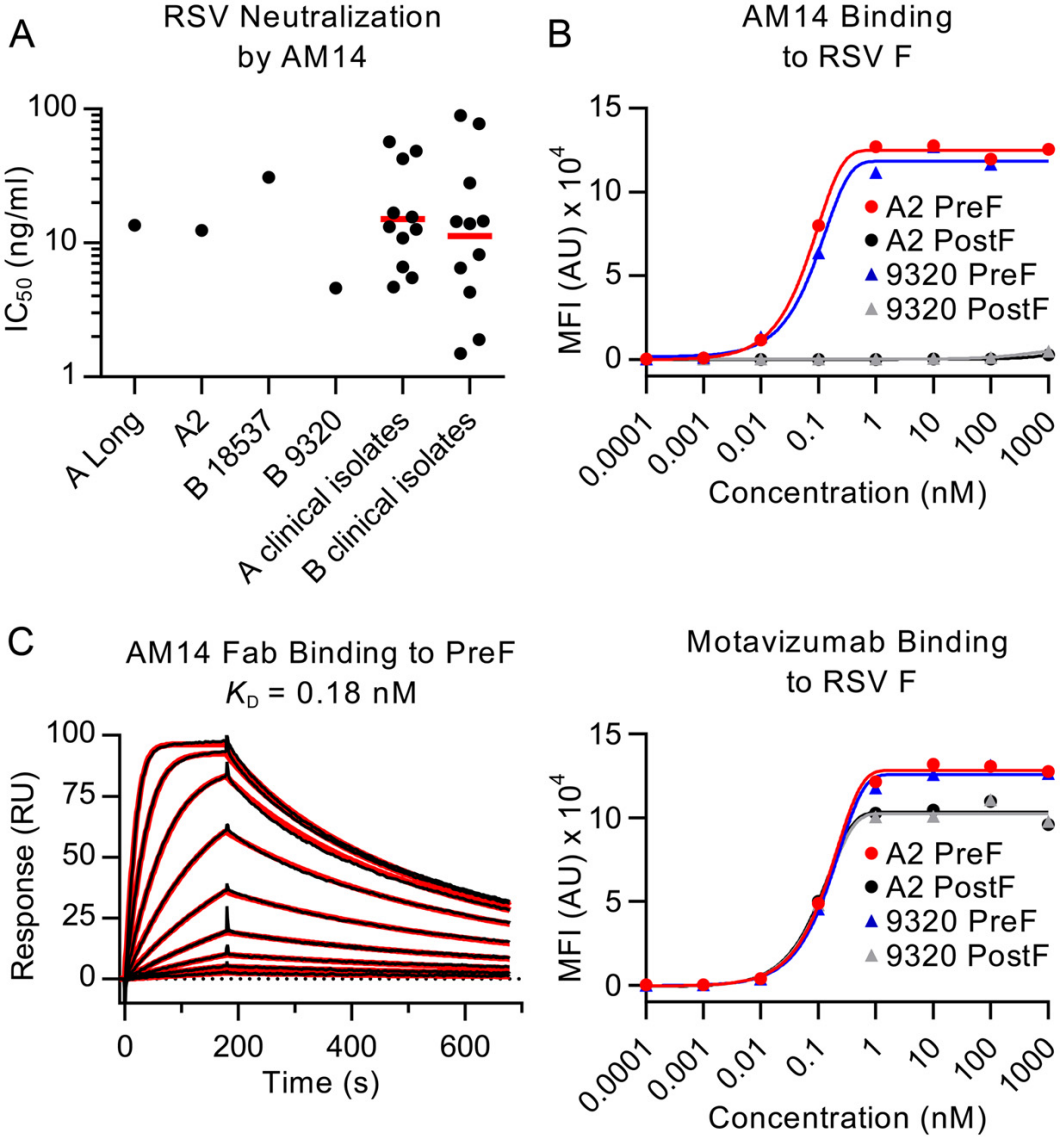
AM14 is a prefusion-specific neutralizing antibody. (A) Neutralization of laboratory strains and clinical isolates of RSV. Red bars are geometric means. (B) Binding of AM14 and motavizumab IgGs to immobilized RSV F proteins was measured using a Luminex system. (C) Binding of AM14 Fab to immobilized prefusion RSV F was measured by surface plasmon resonance. Best fit of the data to a 1:1 binding model is shown in red.

Microneutralization assays were performed to test the ability of AM14 to neutralize infection of HEp-2 cells by various RSV strains. AM14 potently neutralized all RSV strains tested, with IC_50_s of 13.6 ng/ml for strain A Long, 12.4 ng/ml for strain A2, 30.8 ng/ml for subtype B strain 18537 and 4.6 ng/ml for subtype B strain 9320 (Fig 1A). For comparison, palivizumab neutralized these strains with IC_50_s of 300 ng/ml, 320 ng/ml, 380 ng/ml and 120 ng/ml, respectively (S1A Fig). Clinical RSV isolates were also tested in the microneutralization assay (Fig 1A). AM14 neutralized subtype A clinical strains with a geometric mean IC_50_ of 15.1 ng/ml and a range of 4.7–56.9 ng/ml. Neutralization of subtype B clinical strains was similar, with a geometric mean IC_50_ of 11.3 ng/ml and a range of 1.5–89.2 ng/ml. The results obtained in this HEp-2 cell-based assay were similar to those previously reported for neutralization of strain A2 in a Vero cell-based assay (IC_50_ values of 2.1 ng/ml for AM14 and D25, and 209 ng/ml for palivizumab) [23, 28]. Similar to other prefusion-specific neutralizing antibodies tested previously, AM14 did not inhibit attachment of RSV to the surface of HEp-2 cells (S1B Fig) [19], suggesting that it prevents entry by blocking a step downstream of attachment. Collectively, these results demonstrate that AM14 is a potent neutralizer of RSV infection, capable of neutralizing both A and B subtypes equally well by a mechanism independent of viral attachment to the cell surface.

### AM14 is specific for prefusion RSV F

Since the neutralization potency of AM14 was similar to that of the prefusion-specific antibody D25, we hypothesized that AM14 might also exclusively recognize the prefusion conformation. To test this possibility, we performed a Luminex-based binding assay using furin-cleaved RSV F ectodomains stabilized in the prefusion (DS-Cav1) or postfusion (F ΔFP) conformation [18, 26]. In this experiment, AM14 bound tightly to prefusion RSV F derived from A and B subtypes with EC_50_s of 0.63 nM and 0.18 nM, respectively (Fig 1B). In contrast, no binding to furin-cleaved postfusion F was detected. Motavizumab, which binds equally well to both RSV F conformations [18, 19], recognized all proteins tested in this assay, confirming the immobilization of RSV F proteins to the beads (Fig 1B).

To further characterize the binding of AM14 to prefusion RSV F, surface plasmon resonance experiments were performed (Fig 1C). AM14 Fab bound to immobilized prefusion RSV F with an equilibrium dissociation constant (*K*_D_) of 0.18 nM, with rapid association and dissociation rate constants of 1.87 x 10^7^ M^-1^s^-1^ and 3.4 x 10^-3^ s^-1^, respectively. This is in contrast to D25, which bound prefusion RSV F with association and dissociation rate constants more than 10- and 30-fold slower, respectively (1.35 x 10^6^ M^-1^s^-1^ and 9.65 x 10^-5^ s^-1^) (S2A Fig). Therefore, although AM14 binds specifically to prefusion RSV F with sub-nanomolar affinity, its kinetics are much faster than those of other potent antibodies such as D25.

### Structure determination of AM14 in complex with prefusion F

To identify the epitope on prefusion RSV F recognized by AM14, the crystal structure of AM14 alone and in complex with prefusion F was determined. Crystals of AM14 Fab in space group *P*2_1_2_1_2_1_ diffracted X-rays to 2.0 Å, and after a molecular replacement solution was obtained, the structure was built and refined to an *R*_work_/*R*_free_ of 18.6%/22.6% (S3 Fig). To obtain a structure of AM14 Fab bound to RSV F, a matrix of F proteins and Fabs was screened, including ten different protein complexes, eight of which formed crystals. Only four of those diffracted past 8 Å (S1 Table). Prefusion RSV F complexed with both AM14 and motavizumab Fabs formed rod-like crystals in spacegroup *P*2_1_ that diffracted X-rays to 5.5 Å. The asymmetric unit was composed of one prefusion F trimer, three AM14 Fabs and three motavizumab Fabs (S4 Fig). The structure was refined in Phenix with NCS torsion restraints and reference-model restraints to an *R*_work_/*R*_free_ of 21.1%/27.7%. Secondary structures and connecting loops fit the electron density well, particularly at the antibody–F protein interfaces (S5 Fig). Additionally, the electrostatic potential of the interface showed substantial charge complementarity, with a positively charged region on RSV F interacting with negatively charged residues in the complementarity-determining regions (CDRs) of the AM14 Fab (S6 Fig).

**Fig 2:**
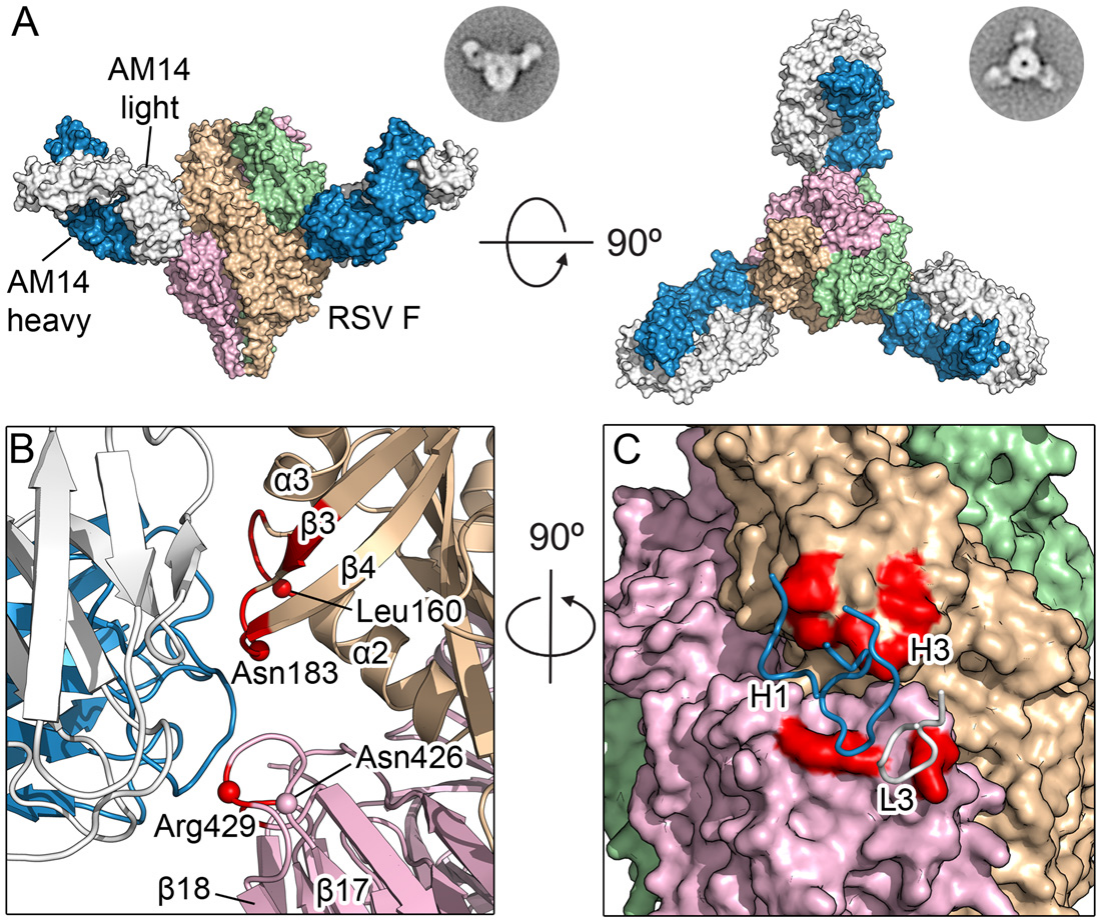
Structures of AM14 in complex with RSV F. (A) Crystal structure of three AM14 Fabs bound to the prefusion RSV F trimer, viewed from the side and the top of RSV F. Negatively stained EM class averages that may correspond to each view are shown in the upper right. AM14 heavy chain is blue, light chain is white, and RSV F protomers are tan, light green and pink (B) Close-up of the side view, colored as in (A). Prefusion RSV F residues with Cα atoms within 8 Å of AM14 Fab Cα atoms are colored red, and Cα atoms of resistance mutations are shown as spheres. (C) Ninety-degree rotation of the view in (B), showing a molecular surface representation of RSV F and the location of the three AM14 CDR loops that contact F. The binding surface spans the two neighboring protomers (tan and pink).

The orientation of the three AM14 Fabs was largely similar to that observed in negative-stain EM images of prefusion RSV F in complex with AM14, with the exception of the side-view, in which the apex of the trimer is not visible (Fig 2A). This difference is likely due to a combination of the trimer apex protruding from the stain and averaging of slightly different tilts of the complex. The crystal structure shows that AM14 binds at the junction of two protomers within the RSV F trimer (Fig 2B), with approximately 330 Å^2^ buried on the membrane-distal protomer and 520 Å^2^ buried on the membrane-proximal protomer. The heavy chain CDRs 1 and 3 make contact with RSV F, as does the light chain CDR 3 (Fig 2C). The AM14 contacts are localized to three regions of the RSV F primary sequence that fold together in the quaternary structure of the prefusion trimer. The first two regions map to the ends of two loops connecting α2 with α3 and β3 with β4, both of which undergo dramatic conformational changes in the pre- to postfusion transition, moving by nearly 100 Å [19]. These two regions together with α4 of antigenic site Ø form the continuous α5 helix of postfusion F, which forms the inner heptad repeat (HRA) of the six-helix bundle, explaining the prefusion-specificity observed for AM14 [19]. The third region of the epitope, located on an adjacent protomer, maps to the loop connecting β17 with β18, which partially overlaps with antigenic site IV and is in a similar conformation in pre- and postfusion structures [18, 19, 22]. Collectively, these data provide a structural basis for the prefusion specificity of AM14, and predict that AM14 is trimer-specific.

### AM14 MARMs map to the three loops identified in the crystal structure

**Fig 3:**
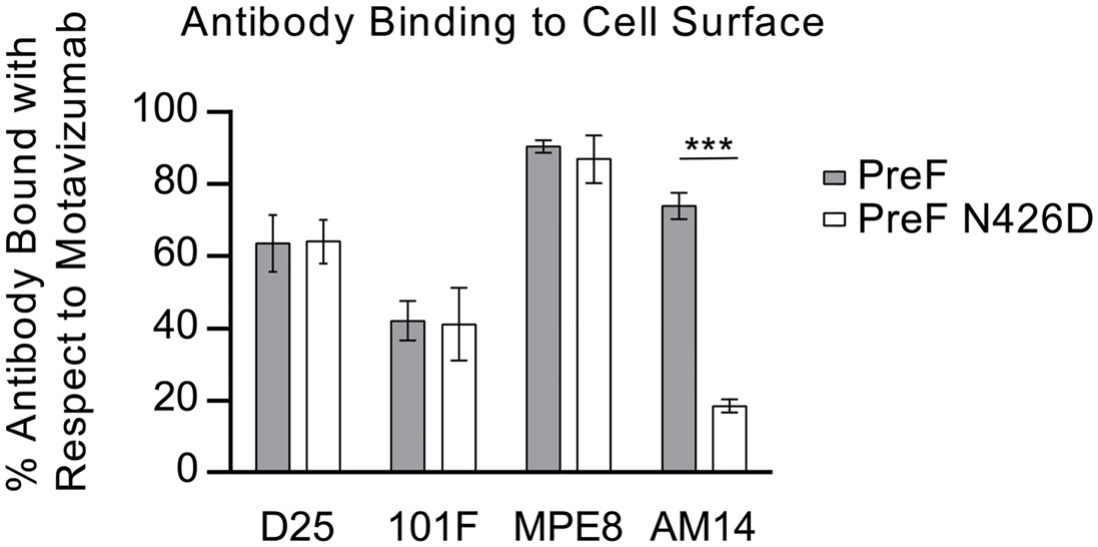
N426D disrupts binding of AM14 to prefusion F. Relative binding of D25, 101F, MPE8 and AM14 to cell surface-expressed prefusion F (grey) or prefusion F containing the AM14 escape mutation, N426D (white) was measured by flow cytometry. Data were normalized to motavizumab binding. Binding of D25, 101F and MPE8 to N426D was comparable to wild-type prefusion F, whereas AM14 binding to N426D was reduced four-fold (*n* = 3, *P* < 0.001; Tukey’s HSD, error bars show standard deviation).

Due to the low resolution of the X-ray crystal structure, we sought to verify the AM14 epitope and identify critical interactions by generating monoclonal antibody-resistant mutants (MARMs). After three rounds of selection in HEp-2 cells, four unique AM14-escape viruses were isolated and sequenced. Three of the viruses each contained a single mutation in RSV F (L160S, N183K, or N426D), whereas the fourth virus contained three mutations in RSV F (I79M/R429S/H515N) (Table 1). Resistance of viruses to neutralization by AM14 was confirmed by microneutralization assays (S7 Fig). For one MARM, N426D, a full-length prefusion F variant was generated and expressed on the surface of HEK293 cells. Binding of AM14 IgG to these cells was reduced approximately four-fold compared to cells expressing wild-type prefusion F (Fig 3). Consistent with this result, binding of AM14 IgG to purified prefusion F N426D was reduced by approximately 100-fold in an ELISA (S8 Fig). Binding of antibody 101F to prefusion F N426D was also slightly reduced when measured by ELISA, which is not surprising given that the 101F epitope (residues 427–437) is close to this region [22]. The binding of motavizumab, MPE8 and D25 was not affected by the N426D mutation, consistent with the known locations of their epitopes.

**Table 1:** Amino acid changes in F protein of AM14 MARMs.

	Amino acid sequence (position number)						
	79	160	183	426	429	514	515
RSV A Long	I	L	N	N	R	H	H
MARM 14 p3	M	-	-	-	S	-	N
MARM 20 p3	-	S	-	-	-	-	-
MARM 21 p3	-	-	-	D	-	-	-
MARM 57 p3	-	-	K	-	-	-	-

Mapping of the MARMs on the prefusion F structure revealed that L160S and N183K are located on the loops connecting α2 with α3 and β3 with β4, respectively, in agreement with the crystal structure (Fig 2B). This verifies that AM14 makes a substantial interaction with the prefusion-specific region of the membrane-distal protomer. Two other MARMS, N426D and R429S, are located on the loop connecting β17 with β18 in the adjacent protomer, near antigenic site IV. The isolation of these MARMs further supports the low-resolution crystal structure and indicates that the membrane-proximal protomer is a critical component of the AM14 epitope. The final two MARMS, I79M and H515N, were part of the same RSV F variant harboring R429S, and are likely compensatory mutations since they are located more than 30 and 80 Å away from the AM14 epitope, respectively.

### AM14 specifically recognizes cleaved, trimeric, prefusion F

**Fig 4:**
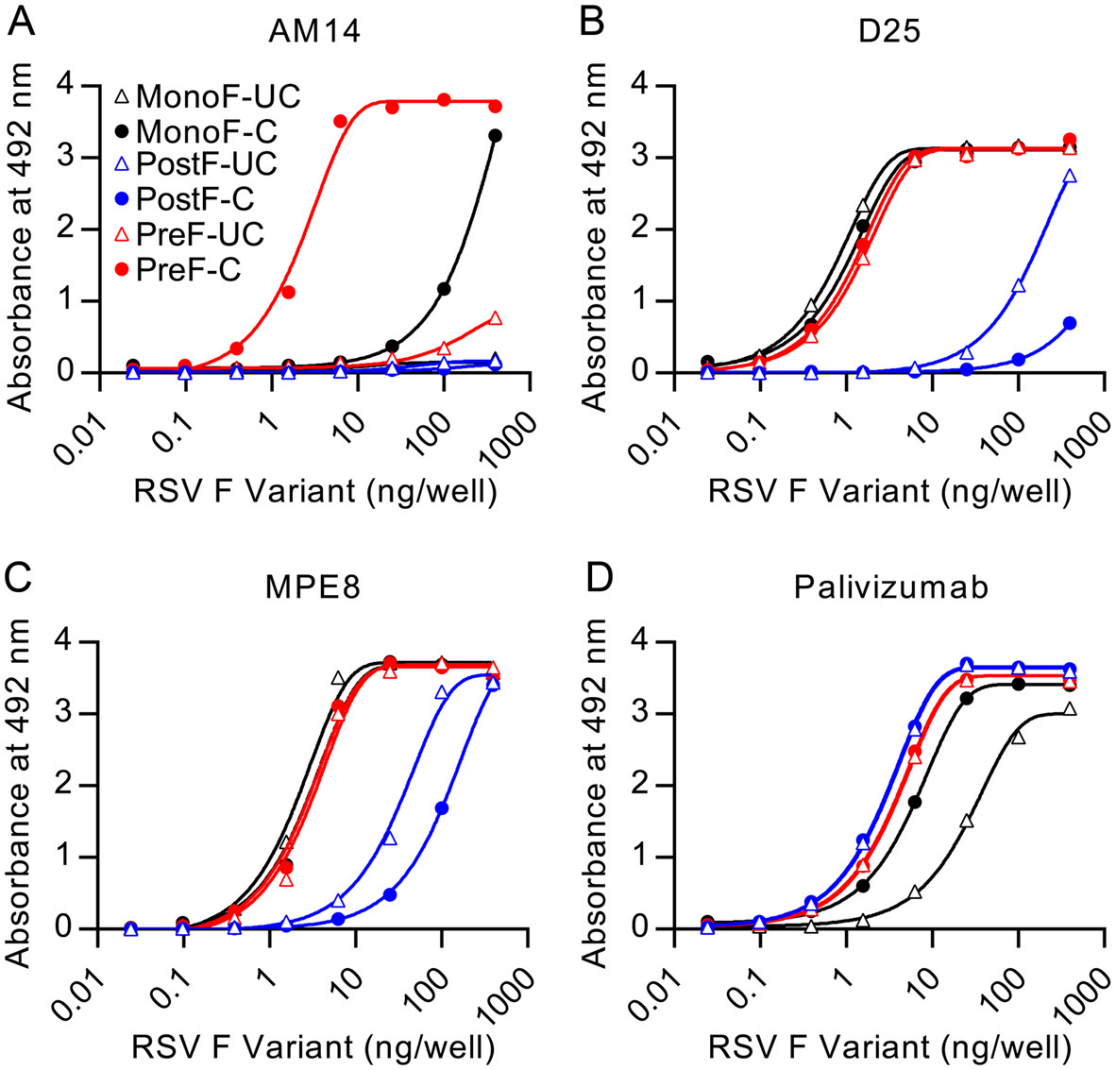
AM14 is specific for cleaved, trimeric RSV F. Binding of antibodies (A) AM14, (B) D25, (C) MPE8, or (D) palivizumab to uncleaved monomeric RSV F (open black triangles), cleaved monomeric RSV F (black circles), uncleaved postfusion RSV F (open blue triangles), cleaved postfusion RSV F (blue circles), uncleaved prefusion RSV F (open red triangles) and cleaved prefusion RSV F (red circles) was measured by ELISA.

The crystal structure and MARMs suggested that AM14 is a quaternary-specific antibody with an epitope spanning two protomers. To test the quaternary specificity, we measured binding of immobilized AM14 to monomeric F and trimeric prefusion F by ELISA (Fig 4). The monomeric F was composed of RSV F residues 1–524 and lacked the foldon trimerization motif. Consistent with the hypothesized quaternary epitope, AM14 binding to monomeric F was reduced by nearly 100-fold compared to the prefusion F trimer (Fig 4A). The residual monomer-binding activity may be due to transient trimerization at high concentrations, which would be indistinguishable from monomeric F binding in this assay.

Since previously described quaternary-specific antibodies against other class I fusion proteins have in some cases shown preference for the mature protease-cleaved fusion protein over the uncleaved protein [29], we sought to determine if cleavage of RSV F was required for AM14 binding. In these assays, AM14 failed to bind prefusion F with mutated furin sites (Fig 4A). In contrast, both D25 and MPE8 (Fig 4B and 4C, respectively) bound to cleaved and uncleaved monomeric and trimeric F proteins with profiles similar to palivizumab, an antibody not expected to have preference for the conformation, cleavage or trimerization of RSV F (Fig 4D). Although D25 was originally described as a quaternary-specific antibody, 90% of its epitope on prefusion F is located on a single protomer, explaining its ability to bind monomeric F (Fig 4B and [19]) and a peptide spanning RSV F residues 153–211 (S2B Fig). The approximately 30-fold decrease in affinity observed for D25 binding to peptide is likely due to the absence of contact residues in the F_2_ subunit and the neighboring protomer, as well as differences in peptide structure compared to the complete prefusion F. Collectively, these results demonstrate that AM14 is unique in its ability to discriminate cleaved, trimeric, prefusion F from the other forms tested.

### Binding of AM14 is sufficient to stabilize RSV F ectodomain in the trimeric prefusion conformation

**Fig 5:**
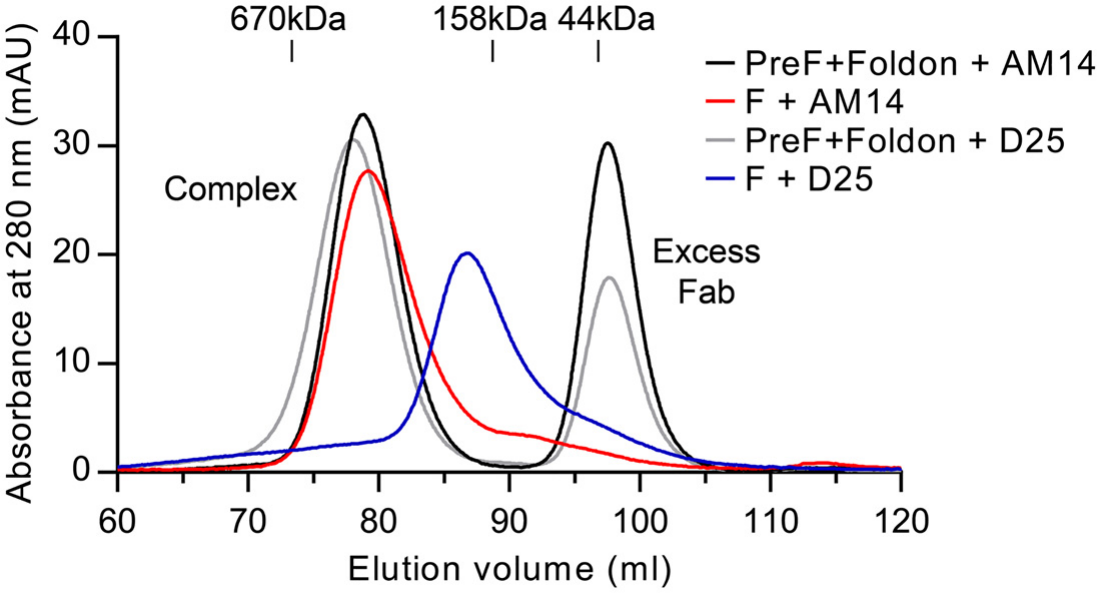
AM14 stabilizes RSV F trimer in the absence of the foldon trimerization motif. Size-exclusion chromatography profiles from a Superose 6 column are shown for AM14 Fab or D25 Fab complexed with prefusion RSV F containing the foldon trimerization motif (black and grey, respectively) and for AM14 Fab or D25 Fab co-expressed with RSV F ectodomain without foldon (red and blue, respectively).

Production of soluble prefusion RSV F has thus far relied on the presence of the foldon motif at the C-terminus of F_1_ to stabilize the weak interprotomer interactions that are normally formed when RSV F is localized in the membrane [19, 26]. Having demonstrated that AM14 is a trimer-specific antibody, we sought to determine if the binding of AM14 was sufficient to stabilize trimeric prefusion F in the absence of a trimerization motif or stabilizing mutations. Since D25 exhibits some degree of interprotomer binding, we also tested this antibody. For this experiment, we first determined the gel filtration elution volumes for purified prefusion, trimeric RSV F (DS-Cav1) incubated with excess Fab. These were then compared to complexes formed by co-expression of each Fab with RSV F ectodomain lacking foldon and stabilizing mutations. Co-expression of AM14 Fab and the F ectodomain resulted in a complex with an elution profile very similar to that of AM14 complexed with DS-Cav1 (Fig 5, compare red and black traces). In contrast, co-expression of D25 and F ectodomain resulted in a complex that eluted later (i.e., smaller) than the corresponding complex of D25 with DS-Cav1 (Fig 5, compare blue and grey traces). Thus, D25 co-expression was not sufficient to stabilize the formation of prefusion F trimer in the absence of a trimerization motif. This property appears to be unique to AM14 due to its epitope being split evenly between two protomers.

## Discussion

AM14 neutralization of RSV is similar in potency to that of the antigenic site Ø antibody D25 [19, 23]. It was originally hypothesized that site Ø-directed antibodies would be the most potent neutralizers due to the location of the epitope on the accessible apex of the prefusion F trimer [19]. Consistent with this hypothesis, the prefusion-specific antibody MPE8, which binds to a lower region on prefusion F, has decreased neutralization potency compared to D25 [25]. In contrast, the potency of AM14 was similar to D25, despite the equatorial binding observed in both the crystal structure and negative stain EM. This demonstrates that antibodies targeting other regions of prefusion F can be as potent as those binding to the apex, which may be an important consideration when optimizing RSV vaccine antigens. In addition, the location of this epitope could make AM14 a candidate for passive prophylaxis. AM14 would have the advantage of high potency and would not block site Ø, leaving this antigenic supersite accessible for inducing protective antibody responses induced by vaccination or infection of the upper airway.

In our ELISAs, prefusion-specific antibodies D25 and MPE8 both bound to uncleaved postfusion F, albeit with low affinity. One possible explanation is that the presence of pep27 in uncleaved postfusion F leads to a more flexible state in which the association of HRA and HRB is weaker and portions of these epitopes that are normally occluded in the cleaved postfusion state are accessible. This hypothesis is supported by the finding that the six-helix bundle-directed antibody 114F bound tighter to cleaved postfusion F (S9 Fig). It is further supported by the reduced SDS-stability of uncleaved compared to cleaved postfusion F trimers. Although uncleaved postfusion F is not biologically relevant, there are a number of subunit vaccines in development that are based upon uncleaved F. Our data suggest that for these proteins in particular, D25 and MPE8 may not be good indicators of prefusion F.

Besides AM14, other antibodies have been identified that recognize higher-order protein structures in both class I and class II viral fusion proteins. The specificity of these antibodies can be grouped into at least three categories. In the first, antibodies contact only one protomer, but recognize a conformation that exists only in the assembled fusion protein, as is the case for the HIV antibody 35O22, which binds to both gp120 and gp41 subunits [30]. This type of antibody has also been proposed for influenza HA antibodies [31]. In the second category, antibodies make direct contact with more than one protomer. This quaternary-specific binding is observed for AM14 and has previously been demonstrated for the influenza antibody HC63 and the HIV antibody PGT151 [29, 32–34]. Additionally, dengue virus antibodies have been identified that recognize both protomers within one dimer of the class II fusion protein E [35]. Interestingly, these antibodies have been reported to shift the monomer–dimer equilibrium, similar to the stabilization of trimeric RSV F that we observed here for AM14 [35]. A third category of higher-order specificity can exist due to the ordered array of glycoproteins on the surface of a virus, as has been described for class II fusion proteins of West Nile virus [36]. Antibody CR4354 binds across two E protein dimers, preventing the rearrangement of E into trimers after exposure to low pH [36]. Thus, the mode of AM14 binding is but one way in which antibodies are able to recognize higher-order structures in class I and II viral fusion proteins.

In addition to quaternary-specific binding, AM14 also exhibited a unique dependence on furin cleavage. RSV F is distinct from the F proteins of other paramyxoviruses due to the presence of two furin sites separated by a glycosylated 27 amino acid spacer, pep27, which is released from the protein after cleavage [15]. There are two likely explanations for the cleavage-dependence of AM14 binding. The first is that pep27 sterically inhibits binding of AM14 to the prefusion trimer. An alternative is that uncleaved F does not adopt the native trimeric state. This would be in contrast to the related paramyxovirus PIV5 F protein, which contains only one cleavage site, since the structures of the cleaved and uncleaved PIV5 prefusion F proteins are nearly identical [37, 38]. Future work on the structure of uncleaved RSV F will be needed to resolve these two possibilities.

The specificity of the potent RSV neutralizing antibody AM14 makes it a useful reagent for probing or isolating the cleaved trimeric state of prefusion F. AM14 may also allow an unparalleled view of prefusion F in its native state, as this antibody can be used to capture trimeric prefusion F without the use of a trimerization motif or stabilizing mutations, as was done for HIV Env using the recently identified trimer-specific antibody PGT151 [29]. AM14 can also be used to help unravel the role of furin cleavage in prefusion F trimerization and to track the order of these events in the secretory pathway, similar to what has been done using antibodies specific for trimeric influenza HA [39, 40]. Further, the high potency and properties of AM14 described here could make it well suited for passive prophylaxis.

## Materials and Methods

### Production of RSV F proteins

Plasmids encoding RSV F prefusion (DS-Cav1) and postfusion (F ΔFP) proteins based on strain A2 [18, 26] were transfected into FreeStyle 293-F cells (Invitrogen). Uncleaved versions of prefusion and postfusion F proteins were produced by changing the basic residues of the two furin-cleavage sites to asparagine residues using site-directed mutagenesis. Proteins were purified from the media using Ni-NTA Superflow resin (Qiagen) and *Strep*-Tactin resin (IBA). Tags were removed by digestion with thrombin or HRV3C protease, followed by gel filtration using a Superose 6 column (GE Healthcare Biosciences). For crystallization, RSV F proteins were expressed in the presence of kifunensine (5 μM), digested with Endo H (10% w/w), mixed with a 1.5-fold molar excess of Fab, and purified using the Superose 6 column. AviTagged F proteins were biotinylated with biotin ligase BirA (Avidity) and separated from excess biotin by gel filtration with a Superdex 200 column (GE).

### Production of IgGs and Fabs

Plasmids encoding antibody heavy and light chains were transfected into Expi293 cells (Invitrogen). IgGs and Fabs were purified using Protein A agarose (Fisher) or CaptureSelect IgG-CH1 affinity matrix (Life Technologies), respectively.

### Luminex binding assay

Biotinylated proteins were coupled to avidin-coated MagPlex beads at 1 μg per 50,000 beads (Radix). Approximately 1000 beads per well were incubated with 10-fold serial dilutions (1 μM to 0.1 pM) of each antibody in a 384-well plate, washed with PBS plus 0.1% BSA with 0.05% Tween 20 and incubated with 0.33 μg/ml phycoerythrin (PE)-conjugated mouse anti-human IgG Fc secondary antibody (Southern Biotech). Beads were washed and emission at 575 nm was measured using the FLEXMAP 3D flow cytometer (Luminex).

### Surface plasmon resonance

Biotinylated DS-Cav1 was immobilized on an SA sensor chip to a total of 292 response units using a Biacore X100 (GE). A buffer-only sample was injected over the DS-Cav1 and reference flow cells, followed by AM14 Fab 2-fold serially diluted from 5 nM to 19.5 pM in HBS-EP+, with a duplication of the 156 pM concentration. The data were double-reference subtracted and fit to a 1:1 binding model using the Biacore X100 analysis software.

### Negative-stain electron microscopy

Samples were diluted to approximately 0.03 mg/ml, adsorbed to a freshly glow-discharged carbon-film grid for 15 sec, and stained with 0.7% uranyl formate. Images were collected semi-automatically using SerialEM [41] on a FEI Tecnai T20 with a 2k x 2k Eagle CCD camera at a pixel size of 0.22 nm/px. Particles were picked automatically and reference-free 2D classification was performed in EMAN2 [42].

### Crystallization and data collection

AM14 Fab crystals were produced by hanging-drop vapor diffusion by mixing 1 μl of AM14 Fab (8.7 mg/ml) with 1 μl of reservoir solution containing 0.1 M sodium acetate pH 4.5, 0.2 M ammonium sulfate and 25% (w/v) PEG 4000. Crystals were soaked in reservoir solution supplemented with 30% (v/v) ethylene glycol and frozen in liquid nitrogen. Data to 2.0 Å were collected at the MacCHESS beamline (Cornell High Energy Synchrotron Source, Cornell University).

The ternary complex was produced by mixing Endo H-treated DS-Cav1 with a 1.5-fold molar excess each of AM14 Fab and motavizumab Fab before separation of the complex from excess Fab by gel filtration. Crystals were produced by hanging-drop vapor diffusion by mixing 0.67 μl of protein (5.6 mg/ml) with 1.33 μl of reservoir solution containing 11.4% (w/v) PEG 8000, 1.7% (v/v) 2-methyl-2,4-pentanediol and 0.1 M imidazole pH 6.5. Many cryopreservation solutions were tested, but diffraction was highest when the crystal was directly plunged into liquid nitrogen after removal of the cold gas layer [43]. Diffraction data were collected to 5.5 Å at the SBC beamline 19-ID (Advanced Photon Source, Argonne National Laboratory).

### Structure determination, model building and refinement

Diffraction data were processed using the CCP4 software suite: data were indexed and integrated in iMOSFLM [44] and scaled and merged with AIMLESS [45]. A molecular replacement solution for the 2.0 Å AM14 Fab dataset was found by PHASER [46] using the heavy and light chains of PDB ID: 4ERS [47] and PDB ID: 4JHA [19], respectively, as search models. The structure was built manually in COOT [48] and refined using PHENIX [49].

A molecular replacement solution for the 5.5 Å ternary complex was obtained using PHASER with prefusion RSV F (PDB ID: 4JHW [19]), motavizumab Fab (PDB ID: 3IXT [11]) and the 2.0 Å AM14 Fab structures as search models. The asymmetric unit contained the prefusion trimer bound by three motavizumab Fabs and three AM14 Fabs. Rigid-body refinement was then performed in PHENIX, and several of the Fab constant domains were manually placed into the electron density using COOT, followed by another round of rigid-body refinement in PHENIX. Group B-factors and coordinates were refined in PHENIX with NCS torsion restraints and reference-model restraints. The reference models were the 2.4 Å prefusion F structure (PDB ID: 4MMS [26]), the 2.75 Å motavizumab Fab structure (PDB ID: 3IXT [11]), and the 2.0 Å AM14 Fab structure determined here. Data collection and refinement statistics for both structures are presented in S2 Table.

### Isolation of MARMs and neutralization assay

RSV strain Long was incubated at 5 x 10^6^ pfu/ml with 3 μg/ml of AM14 for 1 hr prior to infection of confluent HEp2 cells at an MOI of 1.0. Following a 2–3 hour infection, viral inoculum was removed and medium containing 3 μg/ml of antibody was added and incubated at 37°C for 5–7 days. Virus was harvested from wells containing cytopathic effect during the first round of selection and subjected to an additional 2 rounds of selection at 10 μg/ml. Following each round of selection, RNA was isolated from virally infected cells and analyzed by sequencing to determine F protein sequences. After the third round of selection, viruses were plaque purified and microneutralization assays were performed in HEp-2 cells as previously described [50].

### Flow cytometric analysis of prefusion F and N426D variant

A plasmid encoding RSV F residues 1–574 and harboring the DS-Cav1 stabilizing mutations was created, along with an N426D variant, for expression on the cell surface. Expi293 cells were transfected, harvested 48 hours post-transfection, and washed twice with PBS, followed by incubation with RSV F-specific antibodies (1 μg/ml) for 1 hour at 4°C and Alexa488-conjugated goat anti-human secondary antibody (Invitrogen) (5 μg/ml) for 30 minutes at 4°C. Cells were washed, fixed with 0.5% paraformaldehyde, and evaluated by flow cytometry (LSR II instrument, Becton Dickinson). Data were analyzed using FlowJo software (Tree Star) and GraphPad Prism.

### RSV F ELISA

96-well plates were coated with purified monoclonal antibody (AM14, D25, MPE8, palivizumab or 114F) at 6 μg/ml in PBS. Plates were blocked with 2% pig serum in PBS with 0.05% Tween 20 and washed with water. Purified F proteins were serially diluted 4-fold (8 μg/ml to 0.49 ng/ml) and added to plates, which were then washed before incubation with biotinylated anti-HisTag antibody (0.3 μg/ml) (Bio-Rad) and streptavidin-HRP (1:2000) (GE). After addition of *o*-phenylenediamine dihydrochloride (Sigma), reactions were stopped with 2 N sulfuric acid and absorbance was read at 490 nm.

### Analysis of prefusion F complexes by size exclusion chromatography

RSV F ectodomain consisting of residues 1–513 with a C-terminal thrombin cleavage site, 6x His-tag and Strep-tag II was co-expressed with either AM14 or D25 Fab in FreeStyle 293-F cells. Complexes were purified using Ni-NTA and *Strep*-Tactin resins. Separately, DS-Cav1 was produced as described above and mixed with a 1.5-fold molar excess of either AM14 or D25 Fab prior to analysis. Tags were removed from both complexes by thrombin digestion before gel filtration using a Superose 6 XK 16/70 column (GE).

## Supporting Information

S1 Fig AM14 potently neutralizes laboratory strains of RSV but does not inhibit viral attachment to the cell surface. (A) Neutralization of RSV strains A2, Long, 18537 and 9320 were measured using a microneutralization assay. Two-fold serial dilutions of AM14 (red) or palivizumab (black) were incubated with RSV before infection of HEp-2 cells and detection of RSV F on the surface of infected cells by ELISA. In all strains tested, AM14 was greater than one log more potent than palivizumab. (B) A flow cytometry-based attachment assay was modified from a previously described adherent cell assay [22, 51]. Antibodies and heparin were four-fold serially diluted and incubated with RSV A2 prior to attachment to HEp-2 cells and detection of RSV F on the surface of cells by flow cytometry. Although heparin (grey), a known attachment inhibitor, prevented binding of RSV to the cell surface, palivizumab (black) and AM14 (red) did not.
(TIF)

S2 Fig D25 binding to prefusion F and HRA peptide. (A) Binding of D25 Fab to prefusion RSV F was measured by surface plasmon resonance. Prefusion RSV F was immobilized on an SA chip to a total of 300 RU and binding to D25 Fab was measured in 2-fold serial dilutions from 10 to 0.04 nM, with a duplicate of the 0.31 nM concentration. Best fit of the data to a 1:1 binding model is shown in red. D25 Fab bound to immobilized prefusion RSV F with an equilibrium dissociation constant (*K*_D_) of 0.07 nM, with association and dissociation rate constants of 1.35 x 10^6^ M^-1^s^-1^ and 9.65 x 10^-5^ s^-1^, respectively. (B) Binding of D25 to HRA peptide was measured by biolayer interferometry. His-Tagged HRA peptide (RSV A2 F residues 153–211) was immobilized to anti-His sensors and two-fold serial dilutions of D25 IgG from 50 nM to 0.8 nM were assessed for binding on an Octet QK. Red lines are the fit of a global association and then dissociation algorithm in GraphPad Prism, from which the 0.8 nM concentration was excluded. This algorithm was used to calculate an equilibrium dissociation constant (*K*_D_) of 1.9 nM for D25 IgG binding to HRA peptide.
(TIF)

S3 Fig AM14 Fab structure determined to 2.0 Å. Structure of the AM14 Fab with heavy chain shown in blue and light chain shown in white. Complementarity-determining regions (CDRs) are labeled for both the heavy and light chains. The CDR H3 loops of the two Fabs in the asymmetric unit were in different conformations, the second of which is shown in purple. The dotted line indicates a region that was not modeled due to poor electron density. For the complex with RSV F, the Fab with the ordered CDRH3 was used as the search model.
(TIF)

S4 Fig Asymmetric unit of the ternary complex. The asymmetric unit of the ternary complex was composed of one RSV F trimer, three AM14 Fabs and three motavizumab Fabs. The side view and top view of the complex are shown, with RSV F protomers colored tan, light pink and light green. AM14 heavy chains are blue, motavizumab heavy chains are magenta and both AM14 and motavizumab light chains are white.
(TIF)

S5 Fig Main chain model of the 5.5 Å complex fits electron density maps. The main chains are shown as ribbons within the 2Fo—Fc electron density maps for the refined structure (blue), contoured at 1σ. Only one AM14 Fab and the RSV F trimer are shown for clarity. Chains are colored as in S4 Fig. The side view and top view are shown, with zoomed views for the interface between AM14 and the F trimer. The CDR H3 is labeled for both views and fits the electron density well.
(TIF)

S6 Fig Electrostatic potential of the binding interface between RSV F and AM14. Molecular surface representations of prefusion F and AM14 Fab colored according to electrostatic surface potential (red to blue, -8.4 to +8.4 kT/e, respectively). The surfaces are shown in an open-book representation of the binding interface with the approximate footprint for prefusion F and AM14 Fab outlined in yellow and black. A negatively charged region on the AM14 Fab complements a positively charged region on the lower protomer of prefusion F.
(TIF)

S7 Fig AM14-escape variants are not neutralized by AM14. Two-fold serial dilutions of AM14 (red) or palivizumab (black) were incubated with (A) RSV A Long I79M/R429S/H515N, (B) L160S or (C) N183K before infection of HEp-2 cells and detection of RSV F on the surface of infected cells by ELISA. (D) In a separate assay, two-fold serial dilutions of AM14 were incubated with mKate RSV A2 (grey) or mKate RSV A2 N426D (blue) before infection of HEp-2 cells and measurement of fluorescence (excitation of 588 nm and emission at 635 nm) with a plate reader as previously described [52]. All MARMs were neutralized by palivizumab but not by AM14.
(TIF)

S8 Fig N426D disrupts AM14 binding to prefusion F. Plates were coated with (A) stabilized prefusion F or (B) the N426D mutant (1 μg/ml each) and were washed, blocked and incubated with four-fold serial dilutions of motavizumab (black), D25 (blue), 101F (grey), MPE8 (open blue) and AM14 (red) before detection with HRP-conjugated goat anti-human IgG (Santa Cruz Biotechnology) and Super AquaBlue substrate (eBiosciences Inc.). An ELISA plate reader (Molecular Devices, Inc.) was used to read signal at 538 nm. Binding of motavizumab, MPE8 and D25 was similar for both proteins. 101F binding to N426D was slightly reduced, whereas AM14 binding to N426D was reduced approximately 100-fold compared to wild-type prefusion F.
(TIF)

S9 Fig Six-helix bundle-directed antibody 114F shows preference for cleaved postfusion F. (A) Coomassie-stained reducing SDS-PAGE gel of the six proteins used in the ELISAs here and in Fig 4. (B) Binding of 114F, an antibody specific for the six-helix bundle of postfusion F, to the uncleaved monomeric RSV F (open black triangles), cleaved monomeric RSV F (black circles), uncleaved prefusion RSV F (open red triangles), cleaved prefusion RSV F (red circles), uncleaved postfusion RSV F (open blue triangles) and cleaved postfusion RSV F (blue circle) was measured by ELISA. 114F bound more tightly to cleaved postfusion F than to uncleaved postfusion F.
(TIF)

S1 Table Crystals and resulting diffraction for AM14 bound prefusion F complexes. (PDF)

S2 Table Crystallographic data collection and refinement statistics. (PDF)
