# Supplementary figures and images for: Characterization of a Prefusion-Specific Antibody That Recognizes a Quaternary, Cleavage-Dependent Epitope on the RSV Fusion Glycoprotein
Source: PLoS Pathog. 2015 Jul 10;11(7):e1005035. doi: 10.1371/journal.ppat.1005035 (PMC4498696; doi:10.1371/journal.ppat.1005035)

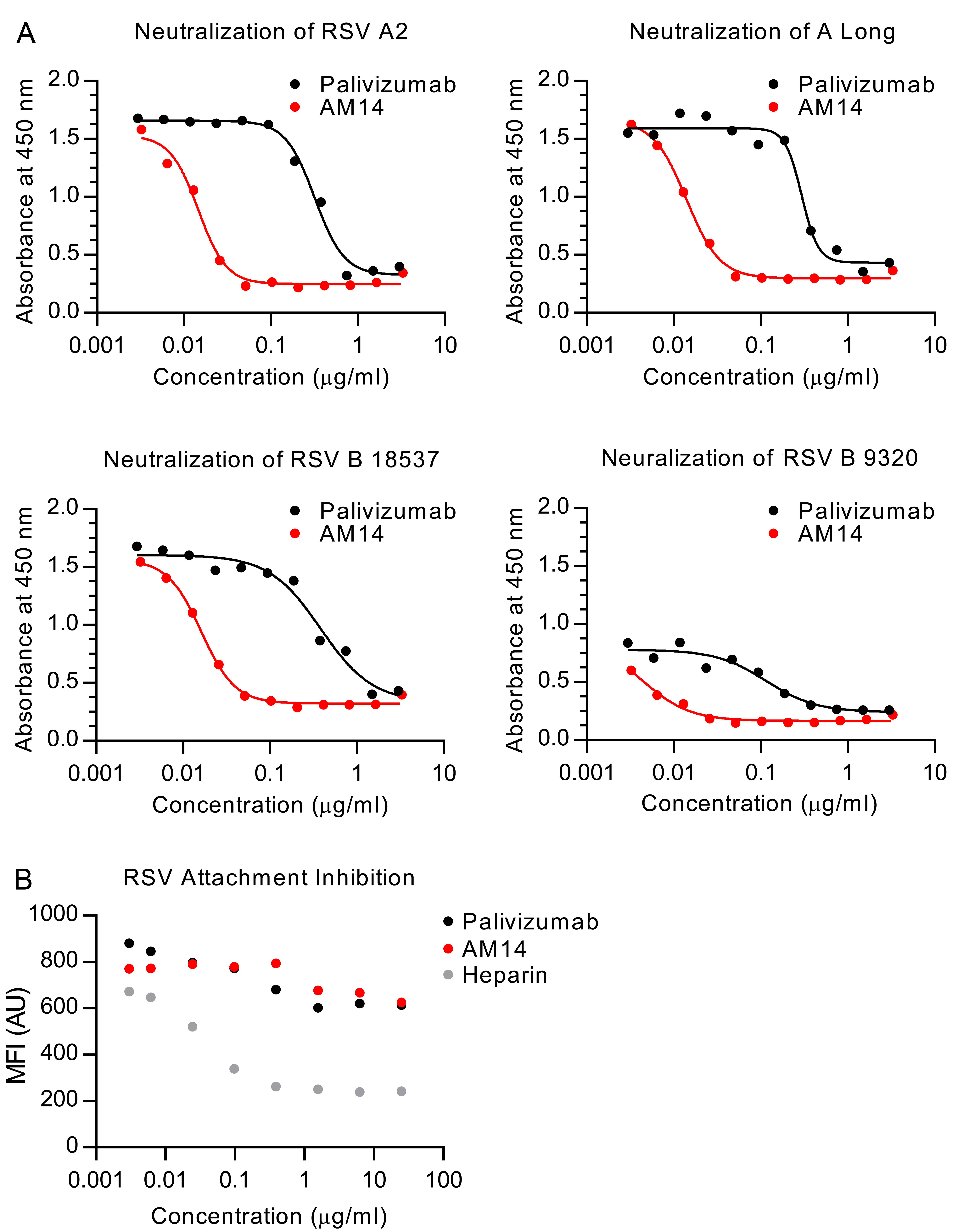

Supplement: S1 Fig — (A) Neutralization of RSV strains A2, Long, 18537 and 9320 were measured using a microneutralization assay. Two-fold serial dilutions of AM14 (red) or palivizumab (black) were incubated with RSV before infection of HEp-2 cells and detection of RSV F on the surface of infected cells by ELISA. In all strains tested, AM14 was greater than one log more potent than palivizumab. (B) A flow cytometry-based attachment assay was modified from a previously described adherent cell assay [22, 51]. Antibodies and heparin were four-fold serially diluted and incubated with RSV A2 prior to attachment to HEp-2 cells and detection of RSV F on the surface of cells by flow cytometry. Although heparin (grey), a known attachment inhibitor, prevented binding of RSV to the cell surface, palivizumab (black) and AM14 (red) did not. (TIF) [file ppat.1005035.s001.tif]

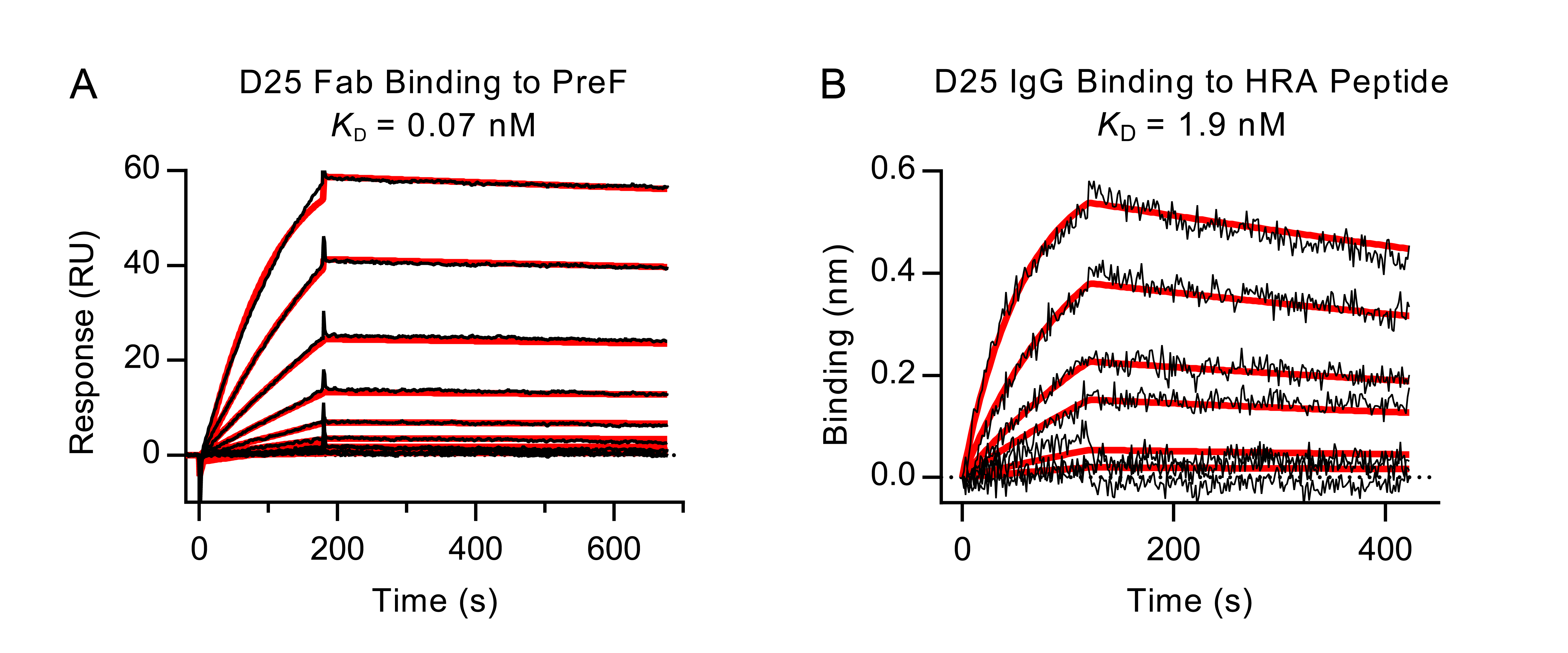

Supplement: S2 Fig — (A) Binding of D25 Fab to prefusion RSV F was measured by surface plasmon resonance. Prefusion RSV F was immobilized on an SA chip to a total of 300 RU and binding to D25 Fab was measured in 2-fold serial dilutions from 10 to 0.04 nM, with a duplicate of the 0.31 nM concentration. Best fit of the data to a 1:1 binding model is shown in red. D25 Fab bound to immobilized prefusion RSV F with an equilibrium dissociation constant (K D) of 0.07 nM, with association and dissociation rate constants of 1.35 x 10^6^ M-1s-1 and 9.65 x 10-5 s-1, respectively. (B) Binding of D25 to HRA peptide was measured by biolayer interferometry. His-Tagged HRA peptide (RSV A2 F residues 153–211) was immobilized to anti-His sensors and two-fold serial dilutions of D25 IgG from 50 nM to 0.8 nM were assessed for binding on an Octet QK. Red lines are the fit of a global association and then dissociation algorithm in GraphPad Prism, from which the 0.8 nM concentration was excluded. This algorithm was used to calculate an equilibrium dissociation constant (K D) of 1.9 nM for D25 IgG binding to HRA peptide. (TIF) [file ppat.1005035.s002.tif]

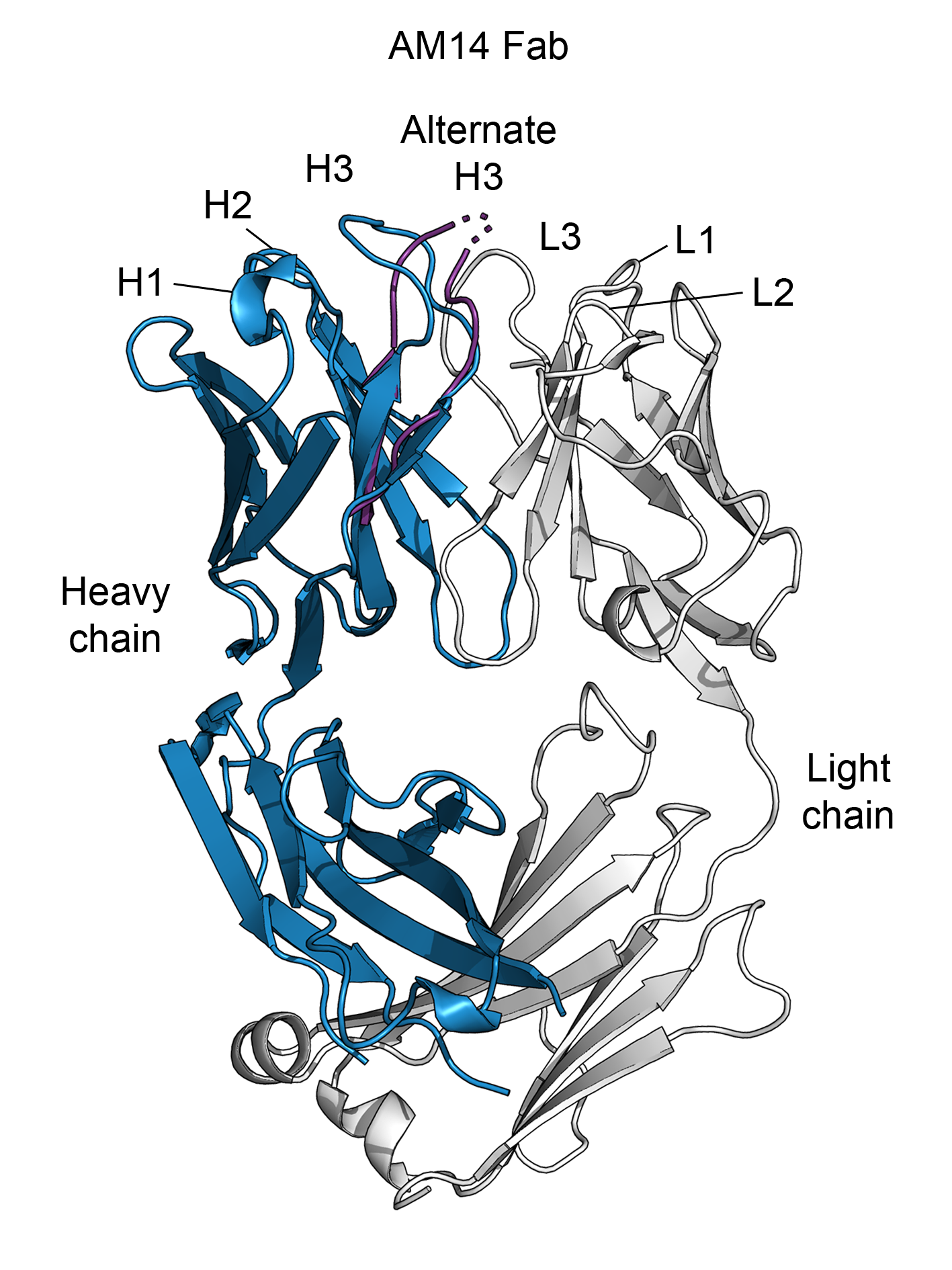

Supplement: S3 Fig — Structure of the AM14 Fab with heavy chain shown in blue and light chain shown in white. Complementarity-determining regions (CDRs) are labeled for both the heavy and light chains. The CDR H3 loops of the two Fabs in the asymmetric unit were in different conformations, the second of which is shown in purple. The dotted line indicates a region that was not modeled due to poor electron density. For the complex with RSV F, the Fab with the ordered CDRH3 was used as the search model. (TIF) [file ppat.1005035.s003.tif]

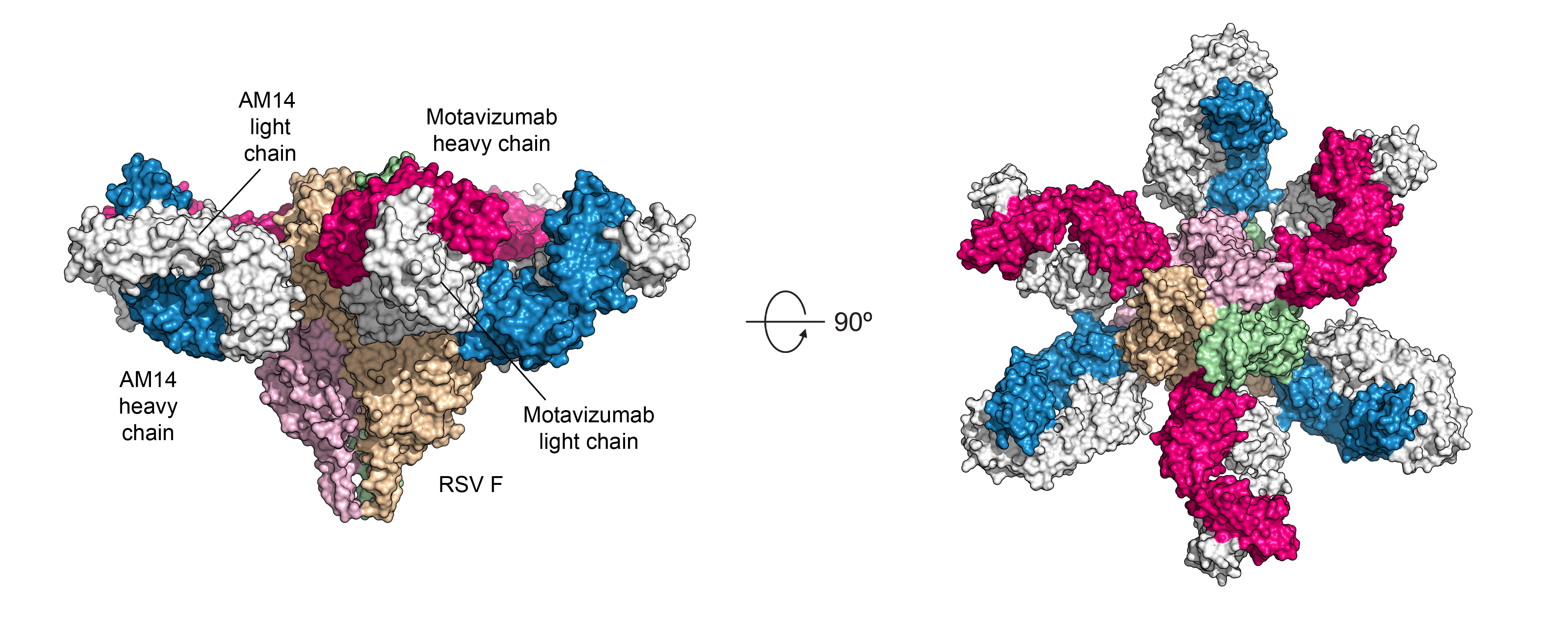

Supplement: S4 Fig — The asymmetric unit of the ternary complex was composed of one RSV F trimer, three AM14 Fabs and three motavizumab Fabs. The side view and top view of the complex are shown, with RSV F protomers colored tan, light pink and light green. AM14 heavy chains are blue, motavizumab heavy chains are magenta and both AM14 and motavizumab light chains are white. (TIF) [file ppat.1005035.s004.tif]

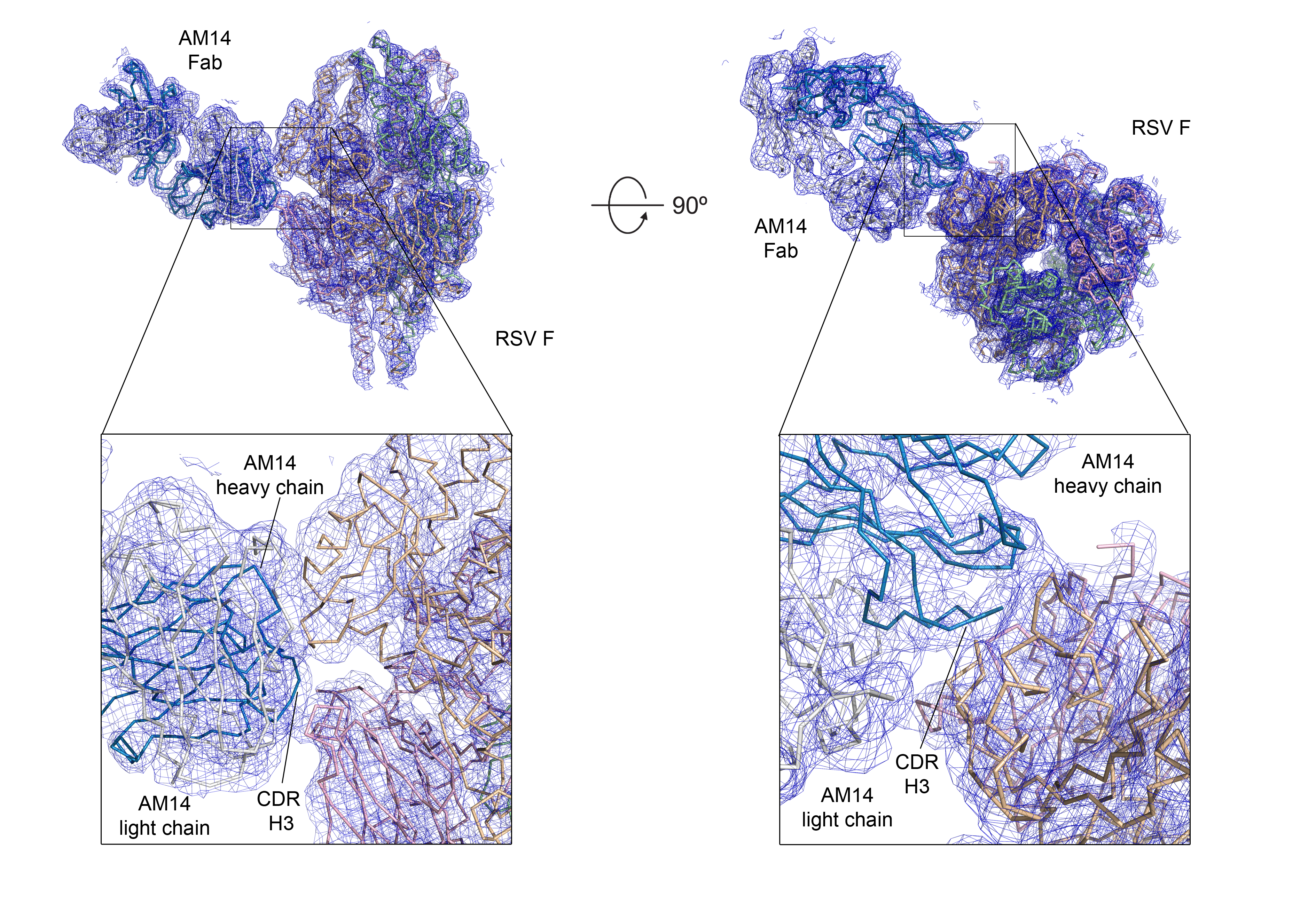

Supplement: S5 Fig — The main chains are shown as ribbons within the 2Fo—Fc electron density maps for the refined structure (blue), contoured at 1σ. Only one AM14 Fab and the RSV F trimer are shown for clarity. Chains are colored as in S4 Fig. The side view and top view are shown, with zoomed views for the interface between AM14 and the F trimer. The CDR H3 is labeled for both views and fits the electron density well. (TIF) [file ppat.1005035.s005.tif]

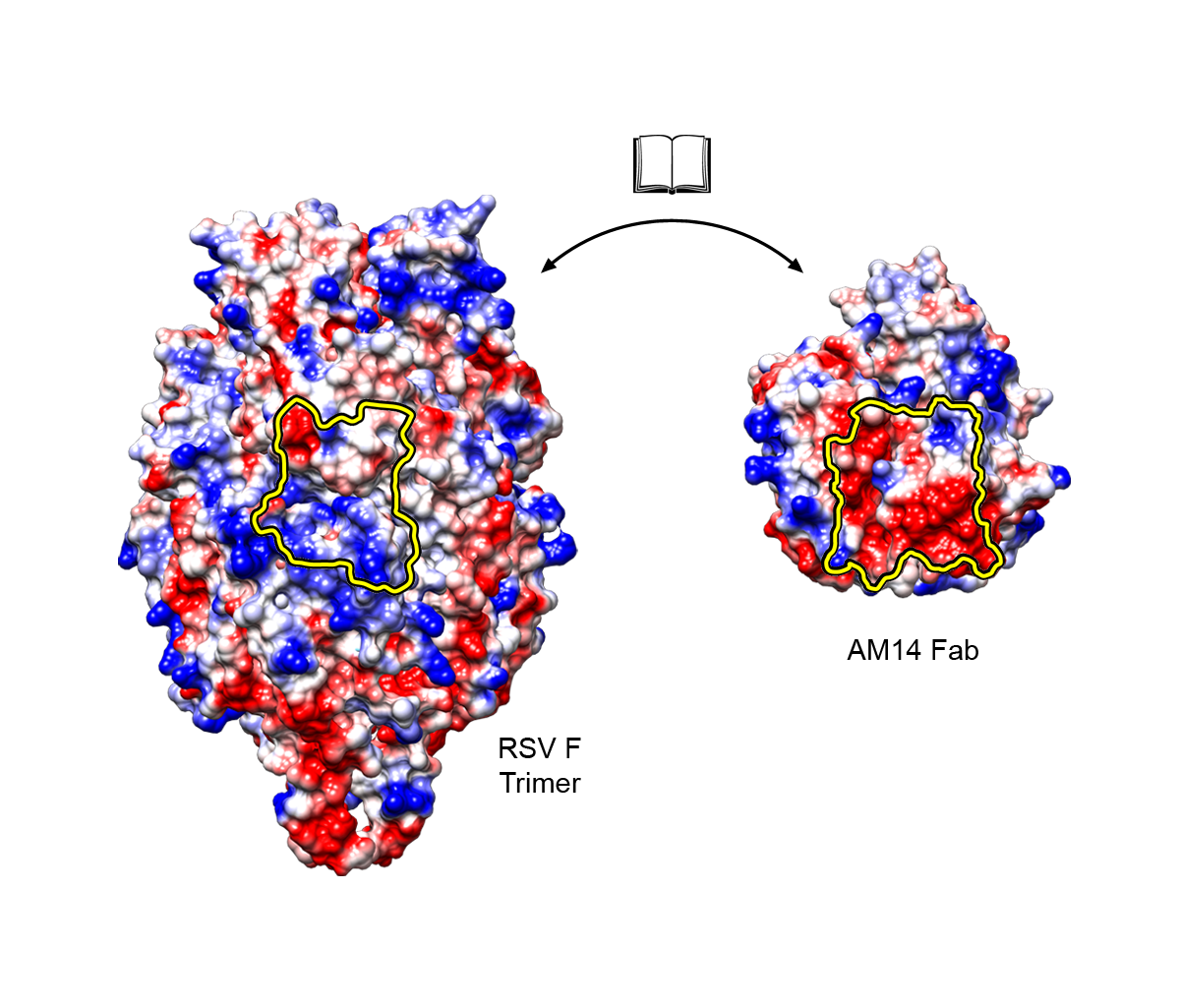

Supplement: S6 Fig — Molecular surface representations of prefusion F and AM14 Fab colored according to electrostatic surface potential (red to blue, -8.4 to +8.4 kT/e, respectively). The surfaces are shown in an open-book representation of the binding interface with the approximate footprint for prefusion F and AM14 Fab outlined in yellow and black. A negatively charged region on the AM14 Fab complements a positively charged region on the lower protomer of prefusion F. (TIF) [file ppat.1005035.s006.tif]

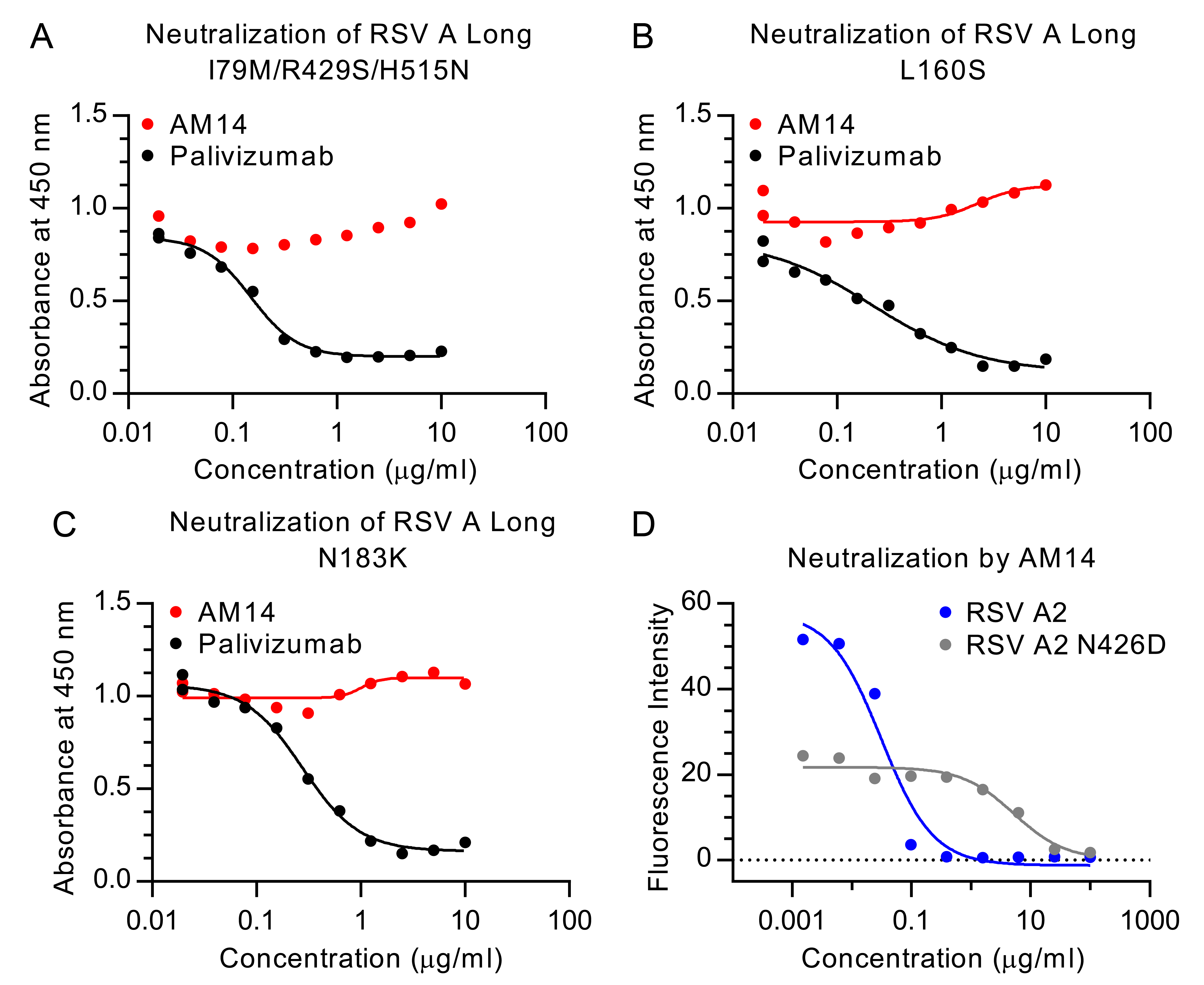

Supplement: S7 Fig — Two-fold serial dilutions of AM14 (red) or palivizumab (black) were incubated with (A) RSV A Long I79M/R429S/H515N, (B) L160S or (C) N183K before infection of HEp-2 cells and detection of RSV F on the surface of infected cells by ELISA. (D) In a separate assay, two-fold serial dilutions of AM14 were incubated with mKate RSV A2 (grey) or mKate RSV A2 N426D (blue) before infection of HEp-2 cells and measurement of fluorescence (excitation of 588 nm and emission at 635 nm) with a plate reader as previously described [52]. All MARMs were neutralized by palivizumab but not by AM14. (TIF) [file ppat.1005035.s007.tif]

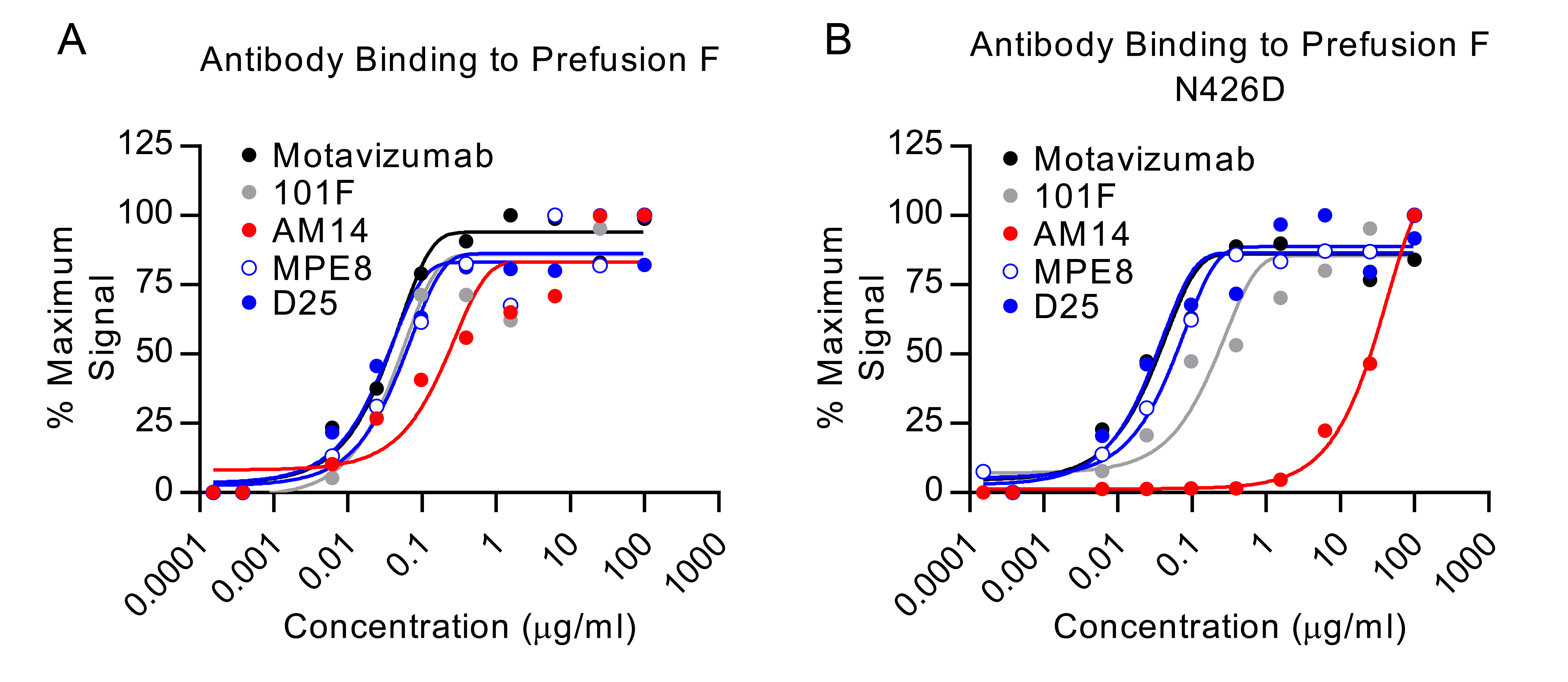

Supplement: S8 Fig — Plates were coated with (A) stabilized prefusion F or (B) the N426D mutant (1 μg/ml each) and were washed, blocked and incubated with four-fold serial dilutions of motavizumab (black), D25 (blue), 101F (grey), MPE8 (open blue) and AM14 (red) before detection with HRP-conjugated goat anti-human IgG (Santa Cruz Biotechnology) and Super AquaBlue substrate (eBiosciences Inc.). An ELISA plate reader (Molecular Devices, Inc.) was used to read signal at 538 nm. Binding of motavizumab, MPE8 and D25 was similar for both proteins. 101F binding to N426D was slightly reduced, whereas AM14 binding to N426D was reduced approximately 100-fold compared to wild-type prefusion F. (TIF) [file ppat.1005035.s008.tif]

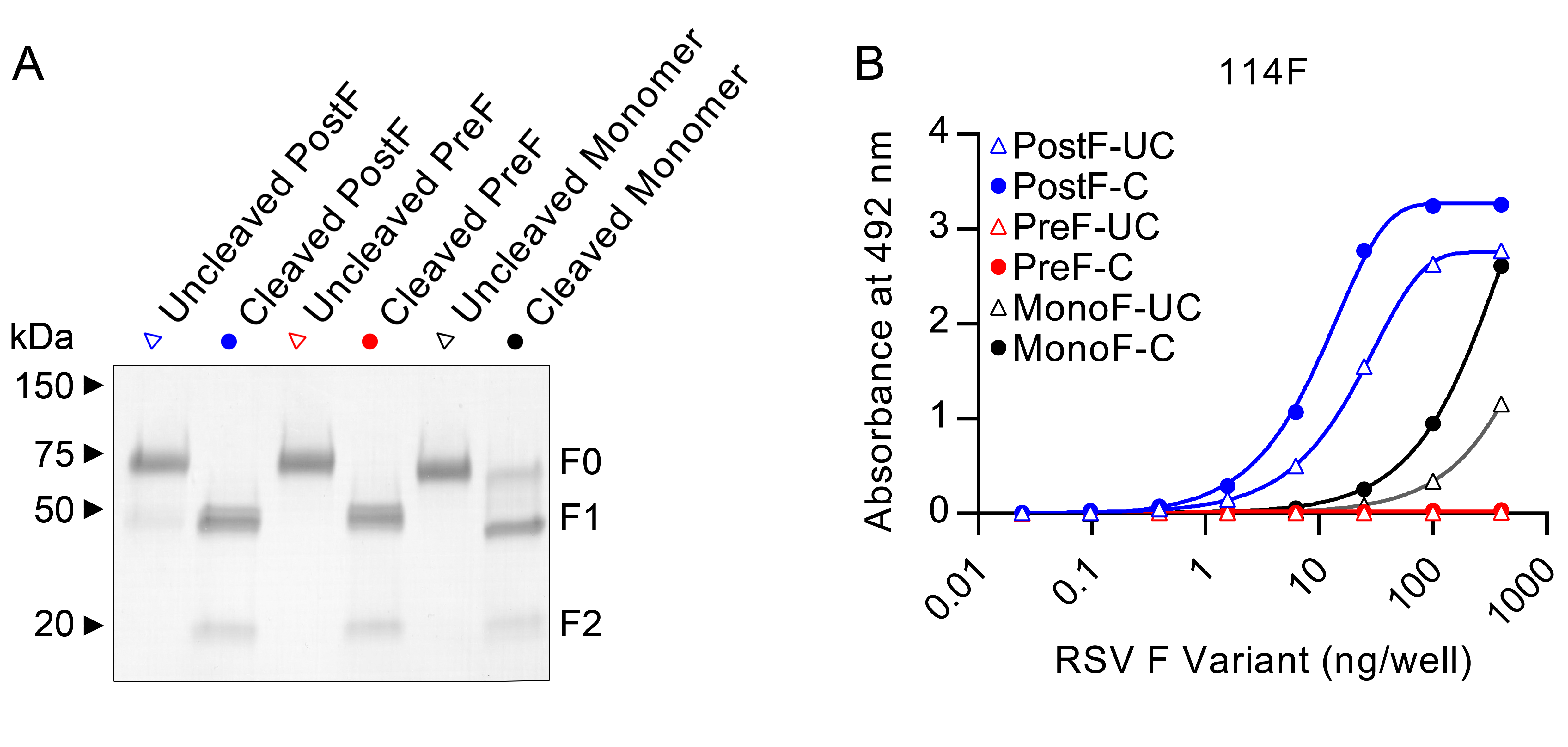

Supplement: S9 Fig — (A) Coomassie-stained reducing SDS-PAGE gel of the six proteins used in the ELISAs here and in Fig 4. (B) Binding of 114F, an antibody specific for the six-helix bundle of postfusion F, to the uncleaved monomeric RSV F (open black triangles), cleaved monomeric RSV F (black circles), uncleaved prefusion RSV F (open red triangles), cleaved prefusion RSV F (red circles), uncleaved postfusion RSV F (open blue triangles) and cleaved postfusion RSV F (blue circle) was measured by ELISA. 114F bound more tightly to cleaved postfusion F than to uncleaved postfusion F. (TIF) [file ppat.1005035.s009.tif]
