# Supplementary material for: Characterization of a Prefusion-Specific Antibody That Recognizes a Quaternary, Cleavage-Dependent Epitope on the RSV Fusion Glycoprotein
Source: PLoS Pathog. 2015 Jul 10;11(7):e1005035. doi: 10.1371/journal.ppat.1005035 (PMC4498696; doi:10.1371/journal.ppat.1005035)
Supplement: S1 Table — (PDF) [file ppat.1005035.s010.pdf]

S1 Table. Crystals and resulting diffraction for AM14 bound prefusion F complexes.

Protein Complex	Crystal Morphology	Diffraction
DS-Cav1 (A2) + AM14	Needles	-
DS-Cav1 (9320) + AM14	Rectangular Plates	5.0 Å Anisotropic
DS-Cav1 (A2) + AM14 + D25	Arrowheads	6.5 Å
DS-Cav1 (9320) + AM14 + D25	Footballs	6.5 Å
DS-Cav1 (A2) + AM14 + Mota	Rods	5.5 Å
DS-Cav1 (A2) + AM14 + Mota + D25	Blocks	17 Å
DS-Cav1 (A2) + AM14 dsFv	Small Diamonds	None
F (A2) without Foldon + AM14	Hexagonal Plates	9 Å
