# Supplementary material for: Characterization of a Prefusion-Specific Antibody That Recognizes a Quaternary, Cleavage-Dependent Epitope on the RSV Fusion Glycoprotein
Source: PLoS Pathog. 2015 Jul 10;11(7):e1005035. doi: 10.1371/journal.ppat.1005035 (PMC4498696; doi:10.1371/journal.ppat.1005035)
Supplement: S2 Table — (PDF) [file ppat.1005035.s011.pdf]

S2 Table. Crystallographic data collection and refinement statistics.

	AM14 Fab	DS-Cav1 + AM14 Fab + Motavizumab Fab
**PDB ID**	4ZYK	4ZYP
**Data collection**		
Space group	$P2_12_12_1$	$P2_1$
Cell constants		
a, *b*, *c* (Å)	69.7, 95.9, 146.0	114.4, 210.3, 118.2
α, β, γ (°)	90, 90, 90	90, 100.5, 90
Wavelength (Å)	0.9770	0.9792
Resolution (Å)	52.6-2.0 (2.05-2.00)	49.6-5.5 (6.15-5.50)
R_{merge}	0.179 (1.190)	0.182 (0.789)
R_{pim}	0.087 (0.615)	0.125 (0.544)
$I / \sigma I$	6.9 (2.2)	4.8 (1.5)
CC(1/2)	0.990 (0.542)	0.939 (0.553)
Completeness (%)	100.0 (99.9)	97.5 (97.6)
Redundancy	5.0 (4.7)	3.0 (2.9)
**Refinement**		
Resolution (Å)	52.6-2.0 (2.03-2.00)	49.4-5.5 (5.84-5.50)
No. reflections	66,725 (2,686)	17,391 (2,886)
R_{work} / R_{free} (%)	18.6/22.6	21.1/27.7
No. atoms		
Protein	6549	29981
Ligand/ion	34	0
Water	623	0
B-factors		
Protein	31.8	217.5
Ligand/ion	51.1	-
Water	39.7	-
R.m.s. deviations		
Bond lengths (Å)	0.003	0.006
Bond angles (°)	0.76	1.35
Ramachandran		
Favored (%)	97.4	94.2
Allowed (%)	2.6	5.3
Outliers (%)	0.0	0.6
